# Construction of a germline-specific RNAi tool in *C*. *elegans*

**DOI:** 10.1038/s41598-019-38950-8

**Published:** 2019-02-20

**Authors:** Lina Zou, Di Wu, Xiao Zang, Zi Wang, Zixing Wu, Di Chen

**Affiliations:** 1 State Key Laboratory of Pharmaceutical Biotechnology and MOE Key Laboratory of Model Animals for Disease Study, Model Animal Research Center, Nanjing University, 12 Xuefu Rd, Pukou, Nanjing, Jiangsu 210061 China; 2 Institute for Brain Sciences, Nanjing University, Nanjing, China

## Abstract

Analysis of complex biological functions usually requires tissue-specific genetic manipulations in multicellular organisms. The *C*. *elegans* germline plays regulatory roles not only in reproduction, but also in metabolism, stress response and ageing. Previous studies have used mutants of *rrf-1*, which encodes an RNA-directed RNA polymerase, as a germline-specific RNAi tool. However, the *rrf-1* mutants showed RNAi activities in somatic tissues. Here we constructed a germline-specific RNAi strain by combining an indel mutation of *rde-1*, which encodes an Argonaute protein that functions cell autonomously to ensure RNAi efficiency, and a single copy *rde-1* transgene driven by the *sun-1* germline-specific promoter. The germline RNAi efficiency and specificity are confirmed by RNAi phenocopy of known mutations, knockdown of GFP reporter expression, as well as quantitative RT-PCR measurement of tissue-specific mRNAs upon RNAi knockdown. The germline-specific RNAi strain shows no obvious deficiencies in reproduction, lipid accumulation, thermo-tolerance and life span compared to wild-type animals. By screening an RNAi sub-library of phosphatase genes, we identified novel regulators of thermo-tolerance. Together, we have created a useful tool that can facilitate the genetic analysis of germline-specific functions in *C*. *elegans*.

## Introduction

The nematode *C*. *elegans* serves as a great model organism in biology research largely due to the ease of genetic manipulations. Genetic screens either by chemical mutagens or RNAi (double-stranded RNA-mediated gene silencing) have led to many discoveries. In *C*. *elegans*, effective RNAi knockdown can be achieved by feeding animals with *E*. *coli* that produce double-stranded (ds) RNAs corresponding to worm genes^1^. Genome-wide RNAi screens have been performed by many labs since the construction of the whole genome RNAi libraries, which are collections of *E*. *coli* strains that produce dsRNAs against nearly every gene in the *C*. *elegans* genome^2^. More focused RNAi screens are also applicable using RNAi sub-libraries of genes that encode transcription factors, chromatin-related factors, kinases, phosphatases and so on.

Numerous studies have demonstrated that multicellular organisms actively use across tissue communications to coordinate biological functions. Thus, tissue-specific genetic manipulations are frequently required to address complex biological questions. Researchers using *C*. *elegans* as a model have developed tools to perform tissue-specific RNAi experiments^3–10^. The strategies usually involve tissue-specific promoters-driving transgene rescue of mutations that are essential for the RNAi machinery. *rde-1*, which encodes an Argonaute protein, functions cell autonomously to ensure RNAi capacity^11^. Therefore, tissue-specific promoters-driving *rde-1* rescue strains will allow RNAi to be effective in a tissue-specific manner.

The *C*. *elegans* germline plays regulatory roles in many biological processes. The germline not only serves as the reproductive tissue that produces gametes, but also affects metabolism, stress response and life span through non-autonomous regulation of gene expression in distal tissues^12–17^. However, the germline tissue is difficult for genetic manipulations since transgenes created by traditional methods are usually silenced in the germline. It was originally reported that mutations in RRF-1, an RNA-directed RNA polymerase, allow RNAi to be effective only in the germline but not in somatic tissues^18^. However, a later study revealed that the *rrf-1* mutants maintain RNAi capacity in the soma, including tissues like the intestine and epidermis^19^. More recently, a strain that carries the *rde-1*(*ne219*) mutation and a single copy *rde-1* transgene driven by the *mex-5* promoter was constructed for tissue-specific RNAi experiments. Since *mex-5* is expressed in both the germline and intestine, this strain shows RNAi to be effective in both tissues^7^.

In order to facilitate the genetic analysis in the *C*. *elegans* germline, we set out to create a tissue-specific RNAi strain that allows RNAi to be effective only in the germline. Through CRISPR/Cas9-based genome editing and Mos1 transposon-based transgenic approaches, we constructed an indel mutation of *rde-1* that carries a single copy *rde-1* transgene driven by the *sun-1* germline-specific promoter. The germline RNAi efficiency and specificity were validated by (1) RNAi phenocopy of known mutations, (2) knockdown of tissue-specific GFP reporter expression via *gfp* RNAi, and (3) quantitative RT-PCR measurement of tissue-specific mRNAs upon corresponding RNAi treatments. Furthermore, the germline-specific RNAi strain shows indistinguishable phenotypes in reproduction, neutral lipid accumulation, thermo-tolerance and life span when compared to wild-type animals. Lastly, we performed an RNAi sub-library screening of phosphatase genes in the germline-specific RNAi strain and identified novel regulators of thermo-tolerance. Together, we have created a useful tool that will help to analyze gene functions in the *C*. *elegans* germline.

## Results

### Construction of a germline-specific RNAi strain via single copy transgenic rescue of an *rde-1* indel mutation in the germline

**Figure 1:**
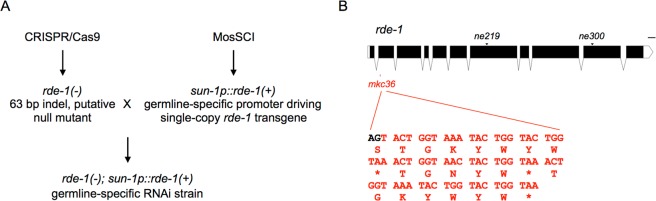
Experimental design of the germline-specific RNAi strain construction. (**A**) Workflow of the experimental design. The *rde-1*(*mkc36*) indel mutation was created using CRISPR/Cas9, and a single copy *rde-1* transgene driven by the *sun-1* promoter was constructed via the MosSCI method. The *rde-1* mutation and germline *rde-1* transgene were then combined by genetic crosses. (**B**) The *rde-1* gene structure. The *ne219* point mutation leads to an E414K missense mutation, whereas the *ne300* point mutation causes a Q825Ochre nonsense mutation. The *mkc36* mutation contains a 67-bp insertion and a 4-bp deletion that created 3 premature stop codons. *Stop codon. Scale, 100 bases. The gene structure was illustrated with the Exon-Intron Graphic Maker tool (http://wormweb.org/exonintron).

In order to study gene functions in the *C*. *elegans* germline, we sought to construct a germline-specific RNAi tool by transgenic rescue of an RNAi machinery mutant *rde-1* (Fig. 1A). Previous studies have applied similar approaches to construct epidermis, muscles and intestine-specific RNAi strains^3,4^. However, the RDE-1 deficiencies were either an E411K missense mutation^3^ or a Q825Ochre nonsense mutation^4^ that is close to the C terminus of the protein. These point mutations may not completely abrogate RDE-1 functions, which could lead to leakiness of RNAi activity in other tissues. To solve this problem, we used CRISPR/Cas9-based genome editing tools to create an *rde-1*(*mkc36*) indel mutation, which carries a 67-bp insertion and a 4-bp deletion in the exon 2 of the *rde-1* coding region (Fig. 1B). Although there is no frame shift in this allele, three premature stop codons (ochre) were introduced to the second exon of *rde-1* (Fig. 1B). RT-qPCR experiments showed that in the *rde-1*(*mkc36*) mutant, the *rde-1* mRNA levels were reduced by around 50% compared to the wild-type N2 animals (Fig. S1). This reduction is likely to be caused by the nonsense-mediated mRNA decay (NMD) triggered by the three premature stop codons. The locations of these stop codons suggest that this indel mutant is a potential null allele.

High copy number-transgenes produced by conventional methods, either in the form of extrachromosomal arrays or integrated, are prone to silencing in the *C*. *elegans* germline. Therefore, we used the Mos1-mediated single copy insertion (MosSCI) method^20^ to make a single copy *rde-1* transgene driven by the germline-specific *sun-1* promoter, which has been shown to be specifically and broadly active in the germline^21,22^. The *rde-1* mutant and transgene were then combined by genetic crosses to create the putative germline-specific RNAi strain DCL569 (*mkcSi13* [*sun-1p::rde-1::sun-1 3′UTR* + *unc-119*(+)] *II; rde-1*(*mkc36*) *V*).

### The germline *rde-1* rescue strain shows robust RNAi capacity in the germline

**Figure 2:**
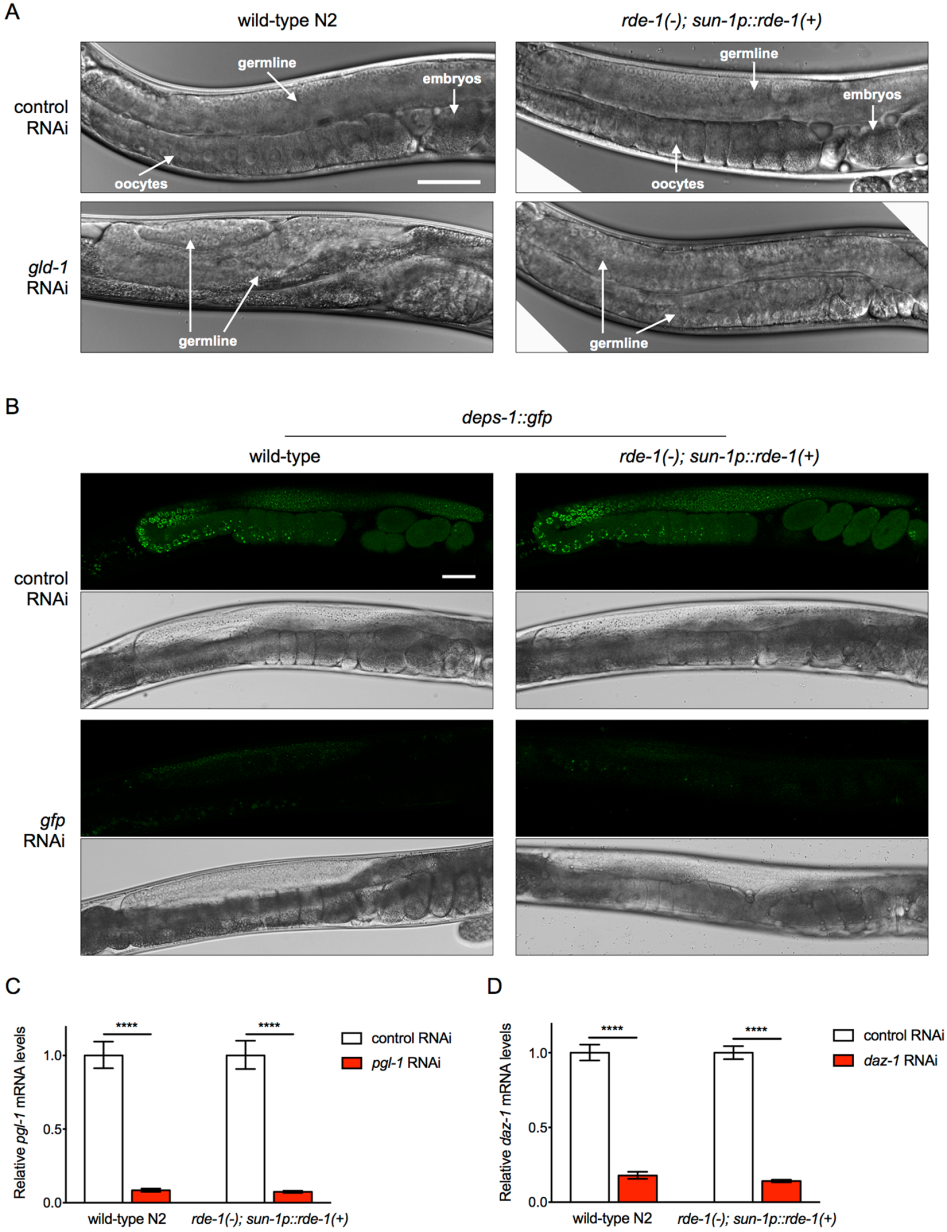
The germline *rde-1* rescue strain shows efficient RNAi knockdown capacity in the germline. (**A**) Representative images of the wild-type N2 and germline *rde-1* rescue strain treated with the control or *gld-1* RNAi. Scale bar, 50 μm. (**B**) Representative fluorescence and bright field images of DEPS-1::GFP in wild-type and germline *rde-1* rescue animals treated with the control or *gfp* RNAi. Scale bar, 50 μm. (**C**,**D**) RT-qPCR measurement of germline genes *pgl-1* (**C**) and *daz-1* (**D**) mRNA levels in wild-type and germline *rde-1* rescue animals treated with the control or corresponding RNAi. ****p < 0.0001 (n = 3, *t* - tests).

To determine whether the *sun-1* promoter driving *rde-1* expression in the germline rescues the RNAi deficiency caused by the *rde-1* mutation, we treated the wild-type N2 and germline *rde-1* rescue strain with *gld-1* RNAi. *gld-1* encodes an RNA-binding protein that functions downstream of the GLP-1/Notch pathway to regulate the mitotic vs. meiotic fates of germline nuclei. Loss-of-function mutations in *gld-1* cause germline nuclei over-proliferation that leads to tumorous germline without oocytes^12,23,24^. Similar to wild-type animals, the germline *rde-1* rescue strain showed germline defects upon the *gld-1* RNAi treatment with around 50% penetrance (Fig. 2A, Table 1). *egg-5* encodes a pseudo-tyrosine phosphatase in oocytes. Inhibition of *egg-5* disrupts oocyte-to-embryo transition and results in lethality^25^. Knockdown of *egg-5* by RNAi effectively caused embryonic lethal phenotype in the wild-type and germline *rde-1* rescue animals (Table 1). Furthermore, the germline *rde-1* rescue strain showed significantly increased germline RNAi efficiency (*p* < 0.01, *t* - tests) compared to the *rrf-1*(−) mutant (Table 1).

**Table 1:** RNAi efficiency in different tissues.

Tissues investigated	Genes targeted by RNAi	RNAi phenotypes	Percent of animals showed RNAi phenotypes (%)^a^				*p* ^b^
			wild type	*rde-1*(−)	*rde-1*(−); *sun-1p::rde-1*(+)	*rrf-1*(−)	
Germline	*gld-1*	Pro, Tum	41.7 ± 1.3	0.0 ± 0.0	52.1 ± 2.7	38.8 ± 1.0	0.0014
Germline	*egg-5*	Emb	85.3 ± 1.7	0.0 ± 0.0	89.8 ± 3.3	45.4 ± 0.9	<0.0001
Intestine	*elt-2*	Gob, Clr	88.6 ± 1.4	0.0 ± 0.0	1.4 ± 1.4^c^	75.9 ± 2.1	<0.0001
Epidermis	*dpy-10*	Dpy	93.5 ± 6.6	0.0 ± 0.0	3.2 ± 2.5^d^	11.1 ± 1.7	0.0097
Epidermis	*tsp-15*	Bli, Dpy	92.2 ± 1.1	0.0 ± 0.0	0.0 ± 0.0	0.0 ± 0.0	/
Muscles	*unc-112*	Prz	98.9 ± 1.1	0.0 ± 0.0	0.0 ± 0.0	0.0 ± 0.0	/
Muscles	*pat-4*	Prz	93.2 ± 2.3	0.0 ± 0.0	0.0 ± 0.0	0.0 ± 0.0	/

^a^Percentage of animals showed RNAi phenotypes. Data are represented as mean ± S.D. based on three independent assays.

^b^*rde-1*(−); *sun-p*::*rde-1*(+) vs. *rrf-1*(−) (t - tests).

^c^*rde-1*(−) vs. *rde-1*(−); *sun-1p*::*rde-1*(+), *p* = 0.1415 (t - test).

^d^*rde-1*(−) vs. *rde-1*(−); *sun-1p*::*rde-1*(+), *p* = 0.0891 (t - test).

Pro: proximal germ cell proliferation abnormal; Tum: tumorous germline.

Emb: embryonic death.

Gob: gut obstructed; Clr: clear.

Dpy: dumpy.

Bli: blistered.

Prz: paralyzed.

We next tested the germline RNAi efficiency by knockdown of germline GFP expression via *gfp* RNAi. DEPS-1 is a P-granule-associated protein that is expressed in the germline^26,27^. DEPS-1::GFP expression is significantly diminished by the *gfp* RNAi treatment in both the wild-type and germline *rde-1* rescue backgrounds (Fig. 2B). Finally, we performed RT-qPCR experiments to test the RNAi efficiency for genes expressed only in the germline, such as *pgl-1*^28^ and *daz-1*^29,30^. Knockdown of *pgl-1* or *daz-1* by RNAi effectively decreased mRNA levels of these genes in the germline *rde-1* rescue strain (Fig. 2C,D). Together, these results demonstrated that RNAi functions effectively in the germline of the DCL569 strain.

### The germline *rde-1* rescue strain shows no obvious RNAi activities in somatic tissues

**Figure 3:**
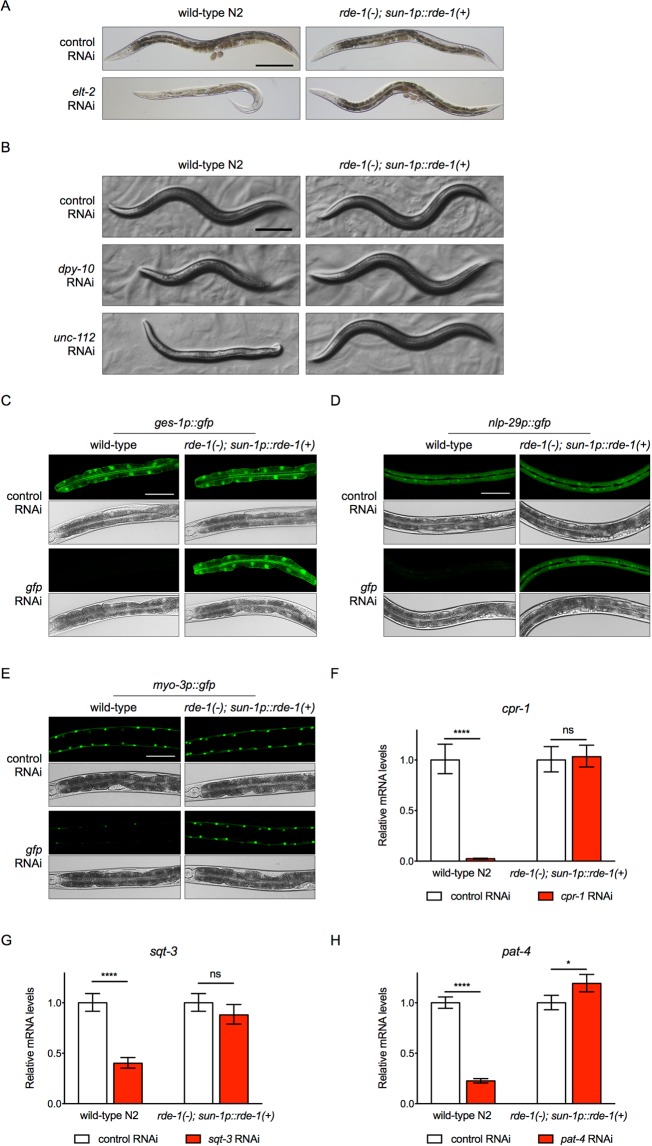
The germline *rde-1* rescue strain shows no obvious RNAi efficiency in somatic tissues. (**A**,**B**) Representative images of the wild-type N2 and germline *rde-1* rescue strain treated with the control or *eft-2* (**A**), *dpy-10* (**B**), *unc-112* (**B**) RNAi. Scale bars, 200 μm. (**C**–**E**) Representative fluorescence and bright field images of the intestinal *ges-1p::gfp* (**C**), epidermal *nlp-29p::gfp* (**D**) and muscular *myo-3p::gfp* (**E**) expression in wild-type and germline *rde-1* rescue animals treated with the control or *gfp* RNAi. Scale bar, 50 μm. (**F**–**H**) RT-qPCR measurement of intestine-specific *cpr-1* (**F**), epidermis-specific *sqt-3* (**G**) and muscle-specific *pat-4* (**H**) mRNA levels in wild-type and germline *rde-1* rescue animals treated with the control or corresponding RNAi. ****p < 0.0001; *p < 0.05; ns, *p* > 0.05 (n = 3, *t* - tests).

The *rrf-1* mutant, which was initially used as a germline-specific RNAi tool^18^, shows leakiness of RNAi effects in the intestine and epidermis^19^. We then tested whether the germline *rde-1* rescue strain shows RNAi leakiness in somatic tissues, including the intestine, epidermis and muscles. We first examined tissue-specific genes, RNAi knockdown of which show morphological phenotypes. Knockdown of ELT-2, an intestinal GATA transcription factor, leads to intestine developmental defects and a clear appearance^19,31^. *dpy-10* encodes a cuticle collagen, RNAi of which results in a Dpy (dumpy, short and fat) phenotype^32^. UNC-112 is a muscle dense body/M-line component. Knockdown of *unc-112* results in paralysis^33^. Unlike wild-type animals, majority of the germline *rde-1* rescue animals showed normal phenotypes upon corresponding RNAi treatments (Fig. 3A,B). Statistical comparisons between the RNAi defective *rde-1*(−) mutant and the germline *rde-1* rescue strain demonstrated that they showed no significant differences (*p* > 0.05, *t* -tests) in resistance to *elt-2* or *dpy-10* RNAi knockdown, whereas the germline *rde-1* rescue strain has significantly less somatic RNAi activities (*p* < 0.01, *t* - tests) compared to the *rrf-1*(−) mutant (Table 1).

We next tested the *gfp* RNAi efficiency using tissue-specific promoter driving GFP reporters. Compared to the wild-type N2, the *gfp* RNAi treatments could not reduce GFP expression produced by the intestinal *ges-1p::gfp*^34^ (Fig. 3C), epidermal *nlp-29p::gfp*^35^ (Fig. 3D) or muscular *myo-3p::gfp*^36^ (Fig. 3E) reporters in the germline *rde-1* rescue strain.

To quantitatively access the RNAi efficiency, we performed RT-qPCR experiments to measure mRNA levels of tissue-specific genes *cpr-1* (intestine)^37^, *sqt-3* (epidermis)^38^ and *pat-4* (muscles)^39^, upon corresponding RNAi treatments. Unlike wild-type animals, the germline *rde-1* rescue strain showed no obvious reduction of the tested mRNAs (Fig. 3F–H). Taken together, these data demonstrate that the germline *rde-1* rescue strain does not allow RNAi to be effective in somatic tissues including the intestine, epidermis and muscles.

### The germline *rde-1* rescue strain shows no obvious deficiencies in reproduction, lipid metabolism, thermo-tolerance and life span

**Figure 4:**
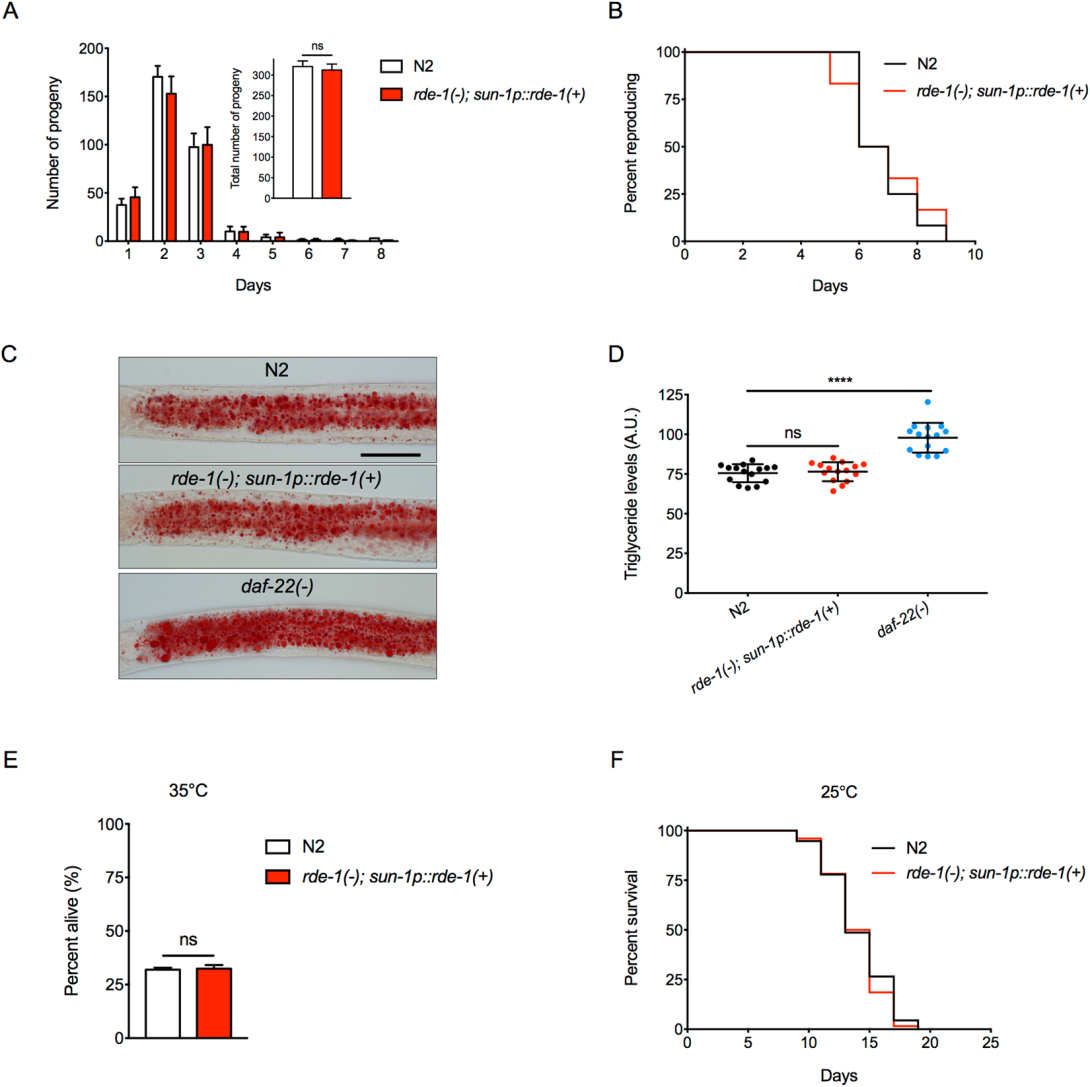
The germline *rde-1* rescue strain has normal reproduction, neutral lipids accumulation, thermo-tolerance and life span. (**A**) Reproductive profile and total brood size of the wild-type N2 and germline *rde-1* rescue strain. Data were represented as mean ± S.D. ns, *p* = 0.1513 (n = 12, *t* - test). (**B**) Reproductive span of the wild-type N2 and germline *rde-1* rescue strain. *p* = 0.8527 (n = 12, log-rank test). (**C**,**D**) Oil Red O staining (**C**) and quantification (**D**) of neutral lipids in the wild-type N2, germline *rde-1* rescue strain and *daf-22* mutant animals. The *daf-22* mutant, which has increased lipid accumulation due to defects in peroxisomal β-oxidation^49^, served as the control. Data were represented as mean ± S.D. ns, *p* = 0.1858, ****p < 0.0001 (n = 15, *t* - tests). Scale bar, 50 μm. (**E**) Survival of the wild-type N2 and germline *rde-1* rescue strain at 35 °C. ns, *p* = 0.6986 (n = 3, *t* - test). (**F**) Survival curves of the wild-type N2 and germline *rde-1* rescue strain at 25 °C. *p* = 0.3543 (log-rank test).

Since the germline *rde-1* rescue strain could be used to study regulatory effects of the germline on development, metabolism and ageing, we examined whether this strain shows normal physiological features. The germline *rde-1* rescue strain has normal reproduction profile, total brood size and reproductive span compared to the wild-type N2 (Fig. 4A,B). Oil Red O staining with fixed animals and quantification indicate that the germline *rde-1* rescue strain has normal neutral lipids levels (Fig. 4C,D). Survival rate of animals treated with heat shock (35 °C for 10 hours) and life span phenotypes are also indistinguishable from those of wild-type animals (Fig. 4E,F). Therefore, the germline *rde-1* rescue strain can be used to study the effects of germline-specific gene knockdown in a variety of assays.

### Identification of novel regulators of thermo-tolerance by an RNAi-based genetic screen

**Figure 5:**
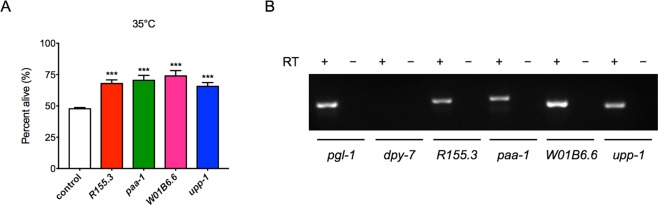
Identification of novel regulators of thermo-tolerance by a germline-specific RNAi screen of phosphatase genes. (**A**) Survival of the *rde-1* germline rescue strain treated with the control, *R155*.*3*, *paa-1*, *W01B6*.*6* or *upp-1* RNAi at 35 °C. ***p < 0.001 (*t* - tests). (**B**) RT-PCR products of *R155*.*3*, *paa-1*, *W01B6*.*6* and *upp-1* amplified from reverse transcription products using RNAs extracted from dissected gonadal tissues. *pgl-1* (germline gene) and *dpy-7* (epidermal gene) serve as positive and negative controls, respectively. RT, reverse transcriptase. The original gel image can be found in Fig. S2.

One important purpose of constructing the germline-specific RNAi strain is to use this tool for RNAi-based genetic screens. As a proof of principle experiment, an RNAi screen was performed to identify novel regulators of thermo-tolerance, since increased intrinsic thermo-tolerance has been associated with the delay of ageing^40^. Phosphatases in many cases play regulatory roles in various signal transduction pathways. However, most of the *C*. *elegans* phosphatase genes have not been well characterized for their biological functions, especially in the tissue-specific context. Thus, we chose an RNAi sub-library containing RNAi clones against 163 phosphatase genes to perform the screen for increased thermo-tolerance in the germline *rde-1* rescue background. After the primary screen and re-tests, we identified four phosphatase genes *R155*.*3*, *paa-1*, *W01B6*.*6* and *upp-1*, RNAi knockdown of which led to significantly increased survival after incubation at 35 °C for 10 hours (>10%, *p* < 0.001) compared to the control RNAi treated animals (Fig. 5A). R155.3 and W01B6.6 are predicted tyrosine phosphatases. PAA-1 is the sole *C*. *elegans* homolog of PR65, a subunit of the protein phosphatase 2A (PP2A) complex^41^. UPP-1 is a uridine phosphorylase, mutations in which result in resistance to the anticancer drug 5-fluorouracil^42^. None of these genes have been associated with heat stress response, especially in a tissue-specific manner.

In order to examine whether the identified genes are expressed in the germline, we performed RT-PCR experiments to detect transcripts of these genes with RNAs extracted from micro-dissected gonadal tissues (Figs 5B, S2). *pgl-1*, a germline gene^28^, and *dpy-7*, an epidermal gene^43^, were used as the positive and negative controls, respectively. Transcripts of *R155*.*3*, *paa-1*, *W01B6*.*6* and *upp-1* can be detected by RT-PCR (Figs 5B, S2), suggesting they are expressed in the germline.

## Discussion

The ease of gene knockdown by RNAi and the existence of whole genome RNAi libraries have enable researchers to perform exploratory studies using *C*. *elegans* as a model. The tissue-specific RNAi strains, which allow RNAi to be effective only in certain tissues, are very useful tools to dissect genes’ functions. The *C*. *elegans* germline has many biological functions. Besides the critical role in reproduction, it can also affect metabolism, stress response and ageing at the whole animal level. In many cases, the regulatory mechanisms involve across tissues, endocrine-like signaling. However, the existing germline-specific RNAi strains all show RNAi efficiency in the soma. Considering the importance of germline in various biological processes, we set out to make a tool that allows RNAi to be effective and specific in the germline.

The Argonaute protein RDE-1, a key component of the RNA-induced silencing complex (RISC), is required for RNAi to be effective^11^. Since RDE-1 functions cell autonomously, tissue-specific transgenic rescue of the *rde-1* mutation will make RNAi to be effective in a spatially restricted manner^3,4,7^. Thus, we made a germline-specific *sun-1* promoter driving *rde-1* single copy transgene to rescue the *rde-1* loss-of-function mutation. Initially, we used the *rde-1*(*ne219*) mutant, which carries an E411K missense mutation, for the strain construction. This mutation has been used to construct epidermis and muscle-specific RNAi strains in previous studies^3^. Further characterization of this strain (DCL484 *mkcSi13* [*sun-1p::rde-1::sun-1 3′UTR* + *unc-119*(+)] *II; rde-1*(*ne219*) *V*) via GFP reporters and RT-qPCR assays revealed that it shows low levels of RNAi capacity in the soma (Fig. S3). We speculated that the leakiness was caused by the residue activities of the *rde-1*(*ne219*) mutation. Thus, we used CRISPR/Cas9 to create an indel *rde-1* mutation and re-constructed the germline-specific RNAi strain (DCL569 *mkcSi13* [*sun-1p::rde-1::sun-1 3′UTR* + *unc-119*(+)] *II; rde-1*(*mkc36*) *V*). Further analyses of this strain demonstrated that it does not show obvious RNAi activities in the soma in various assays (Fig. 3, Table 1). However, we cannot completely rule out the possibility that the differences in somatic RNAi activities between the DCL484 and DCL569 strains were caused by secondary mutations in the backgrounds, although both *rde-1*(*mkc36*) and *rde-1*(*ne219*) mutants have been backcrossed with the wild-type N2 for multiple times.

In this study, we examined the DCL569 germline *rde-1* rescue strain as a potential germline-specific RNAi tool by testing the RNAi capacity in the germline, intestine, muscles and epidermis, which are the most commonly tested ones in tissue-specific studies by the *C*. *elegans* research community. However, there are tissues such as the pharynx, somatic gonad, coelomocytes and so on, which have not been fully tested in this strain. RT-qPCR assays demonstrate that somatic gonadal genes, such as *rin-1* and *lag-2* cannot be effectively suppressed by RNAi in the germline *rde-1* rescue strain (Fig. S4). Nevertheless, more experiments are required to determine whether RNAi capacity is completely restricted to the germline tissue of the DCL569 strain.

Besides RNAi knockdown, researchers in the community have created other tools to achieve tissue-specific genetic manipulations. For example, researchers have adapted the auxin inducible degradation (AID) system in *C*. *elegans*^44^. AID allows drug-inducible, tissue-specific depletion of proteins. However, it requires knock-in of a Degron encoding sequence to the genes of interests via CRISPR/Cas9, which makes this technique more appropriate for focused studies rather than large-scale genetic screens. The germline-specific RNAi strain created in this study is useful for high throughput genetic studies. A pilot RNAi screen of phosphatase genes for thermo-tolerance phenotypes were performed, and several novel heat stress resistance regulators were identified. These results demonstrate that the germline-specific RNAi tool that we constructed can be used for genetic screens to analyze novel functions of germline genes.

## Methods

### *C*. *elegans* strains and maintenance

Strains were cultured on NGM agar plates seeded with *E*. *coli* OP50 at 20 °C unless otherwise stated. The following *C*. *elegans* strains were obtained from the Caenorhabditis Genome Center:

Bristol (N2) strain as the wild-type strain

CB7272 *ccIs4251* [(*pSAK2*) *myo-3p::GFP::LacZ::NLS* + (*pSAK4*) *myo-3p::mitochondrial GFP* + *dpy-20*(+)] *I; mIs12* [*myo-2p::GFP* + *pes-10p::GFP* + *F22B7*.*9p::GFP*] *II; dpy-17*(*e164*) *III; frIs7* [*nlp-29p::GFP* + *col-12p::DsRed*] *IV; uIs69* [*pCFJ90*(*myo-2p::mCherry*) + *unc-119p::sid-1*] *V*

EG4322 *ttTi5605 II; unc-119*(*ed9*) *III*

JH3207 *deps-1*(*ax2063*[*deps-1::GFP*]) *I*

MAH23 *rrf-1*(*pk1417*) *I*

SJ4144 *zcIs18* [*ges-1::GFP*(*cyt*)].

The following strains were generated in D.C. lab:

DCL455 *mkcSi13* [*sun-1p::rde-1::sun-1 3′UTR* + *unc-119*(+)] *II; unc-119*(*ed9*) *III*

DCL484 *mkcSi13* [*sun-1p::rde-1::sun-1 3′UTR* + *unc-119*(+)] *II; rde-1*(*ne219*) *V*

DCL565 *rde-1*(*mkc36*) *V*

DCL569 *mkcSi13* [*sun-1p::rde-1::sun-1 3′UTR* + *unc-119*(+)] *II; rde-1*(*mkc36*) *V*

DCL582 *mkcSi13* [*sun-1p::rde-1::sun-1 3′UTR* + *unc-119*(+)] *II; frIs7* [*nlp-29p::GFP* + *col-12p::DsRed*] *IV; rde-1*(*mkc36*) *V*

DCL590 *ccIs4251* [(*pSAK2*) *myo-3p::GFP::LacZ::NLS* + (*pSAK4*) *myo-3p::mitochondrial GFP* + *dpy-20*(+)] *I; mkcSi13* [*sun-1p::rde-1::sun-1 3′UTR* + *unc-119*(+)] *II; rde-1*(*mkc36*) *V*

DCL592 *deps-1*(*ax2063*[*deps-1::GFP*]) *I; mkcSi13* [*sun-1p::rde-1::sun-1 3′UTR* + *unc-119*(+)] *II; rde-1*(*mkc36*) *V*

DCL593 *mkcSi13* [*sun-1p::rde-1::sun-1 3′UTR* + *unc-119*(+)] *II; rde-1*(*mkc36*) *V; zcIs18* [*ges-1::GFP*(*cyt*)].

### Molecular cloning

The *sun-1* promoter driving *rde-1* rescue plasmid was constructed by cloning PCR fragments of the 468-bp *sun-1* promoter, 3572-bp *rde-1* genomic sequence with all the exons and introns, and 780-bp *sun-1* 3′UTR into the MosSCI vector pCFJ151 (Addgene #71720) using the NEBuilder HiFi DNA Assembly Cloning Kit (NEB). Sequences of primers were shown in Table S1.

The *rde-1* knockout plasmids were generated by inserting targeted sgRNA fragments into the pDD162 vector (Addgene #47549) via the site-directed mutagenesis kit (TOYOBO). The sgRNAs were designed using the CRISPR DESIGN tool (http://crispr.mit.edu).

sgRNA 1 sequence: TTATCGTCATTCTCTCGATC

sgRNA 2 sequence: AGGCCCACTGGTAAATGCGA.

### Generation of the *rde-1* indel mutation by CRISPR/Cas9

The *rde-1*(*mkc36*) indel mutation was generated via CRISPR/Cas9-based genome editing approach^45^. A DNA mix containing the Cas9-sgRNA plasmids (50 ng/µl) and selection marker pCFJ90 P*myo-2*::mCherry (2.5 ng/µl) was injected into N2 young adults. Animals from the F1 generation were screened by PCR for insertions and/or deletions. The *rde-1*(*mkc36*) homozygous mutant was identified from the F2 generation by PCR and the mutations were verified by DNA sequencing of PCR products.

### Single copy transgene by MosSCI

The *sun-1p::rde-1* transgenic strain was constructed by injection of a DNA mix containing 37.5 ng/µl targeting plasmid, 50 ng/µl pCFJ601 (P*eft-3*::Mos1 Transposase), 10 ng/μl neuronal selection marker pGH8 (P*rab-3*::mCherry) and 2.5 ng/μl pharyngeal selection marker pCFJ90 (P*myo-2*::mCherry) into the EG4322 strain (*ttTi5605 II; unc-119*(*ed9*) *III*) according to the previously described protocol^20^. After injection, worms were maintained at 25 °C until starved. Single non-Unc worms without the selection markers were spread to new plates. Successful insertions were confirmed by PCR and DNA sequencing.

### RNAi by feeding

*E*. *coli* strains that carry either the empty vector L4440 (control) or various gene-targeting constructs were cultured and induced for dsRNA production as previously described^46^. For RNAi treatments, gravid adult worms were allowed to lay eggs on RNAi plates at 20 °C for 2 hours. Synchronized animals were collected for various assays at the L4 larval stage or day 1 of adulthood. All RNAi clones were verified by DNA sequencing.

### Microscopy and imaging

Animals were immobilized in 5 μl of 1% sodium azide on 2% agarose pads. GFP fluorescence and the corresponding bright field images were taken using a Zeiss LSM880 confocal microscope under non-saturating conditions. Images shown in the same panel were taken with the same exposure time and adjusted with identical parameters using the Adobe Photoshop.

### Reproduction profile

L4 larvae were individually placed onto NGM plates and then transferred to new plates every 24 hours at 20 °C. Numbers of progeny on each plate were counted 2 days later after removing the adult animals.

### Lipid staining by Oil Red O

Oil red O staining was performed as previously described^47^. L4 larvae were collected and fixed in 1% formaldehyde and frozen at −80 °C. The samples were frozen in dry ice/ethanol bath and thawed under a stream of warm water for three cycles. After washing twice with the S buffer, worms were incubated with the Oil red O (3 mg/ml) solution for 30 minutes at the room temperature. Animals were then washed with the S buffer and incubated on ice for 15 min. Images were taken with a Nikon Eclipse Ni-U microscope and DS-Fi2 color CCD. Triglyceride levels of the second pair of intestinal cells were quantified using the ImageJ software.

### Thermo-tolerance

Synchronized day 1 adult worms were incubated at 35 °C for 10 hours before counting the numbers of alive or dead animals. About 80–100 worms were used in each experiment and each assay was repeated for three times.

### Life span

Worms at the late L4 stages were transferred to fresh NGM plates and incubated at 25 °C for survival. FUdR (20 μg/ml) was added onto NGM plates during the reproduction period to prevent progeny production. Animals were scored as alive, dead (no response to gentle touch) or lost (death from non-ageing causes) every other day. Survival curves were plotted with the GraphPad Prism 6 software and statistical analyses were performed using the log-rank method.

### RNAi screen

An RNAi sub-library that contains RNAi clones against 163 predicted phosphatase genes was used to identify regulators of thermo-tolerance. Synchronized DCL569 (*mkcSi13* [*sun-1p::rde-1::sun-1 3′UTR* + *unc-119*(+)] *II; rde-1*(*mkc36*) *V*) L1 larvae were transferred onto the seeded RNAi plates and incubated at 20 °C until animals reached the day 1 adulthood. In the primary screen, RNAi-treated animals were incubated at 35 °C for 12 hours until the control RNAi treated animals were all dead. Plates with live animals were regarded as thermo-tolerant candidates and were re-tested in the secondary screen. Candidates that passed two rounds of tests were used in the final assays, in which RNAi-treated animals were incubated at 35 °C for 10 hours, and the survival percentages were compared with the control RNAi-treated animals.

### RT-qPCR

About 600 synchronized day 1 adult worms were collected and frozen in the Trizol reagents (Takara). Total RNAs were extracted using the Direct-zol RNA mini prep kit (ZYMO Research) and the cDNAs were synthesized using the reverse transcription system (Takara). Quantitative PCR reactions were performed in triplicates on a Roche LightCycler 480 real-time PCR machine using the SYBR Green dye (Takara). Relative gene expression levels were calculated using the 2^−ΔΔCt^ method and plotted as median with range^48^. RT-qPCR experiments were performed at least three times with independent RNA extractions. Primer sequences can be found in Table S1.

### RT-PCR using dissected gonadal tissues

L4 larvae were transferred into the S buffer (100 mM sodium chloride and 50 mM potassium phosphate [pH 6.0]) on a glass slide. Heads of animals were cut off near the pharynx using syringe needles to allow the gonad to pop out for collection. About 40–50 gonadal tissues were collected in the Trizol reagents for total RNA extraction. Primer sequences can be found in Table S1.

## Supplementary information

Supplemental information
