# Supplementary material for: Construction of a germline-specific RNAi tool in C. elegans
Source: Sci Rep. 2019 Feb 20;9:2354. doi: 10.1038/s41598-019-38950-8 (PMC6382888; doi:10.1038/s41598-019-38950-8)
Supplement: Supplementary file 1 — Supplemental information [file 41598_2019_38950_MOESM1_ESM.pdf]

Construction of a germline-specific RNAi tool in *C. elegans*

Lina Zou[1], Di Wu[1], Xiao Zang[1], Zi Wang[1], Zixing Wu[1] and Di Chen[1, 2]

[1]State Key Laboratory of Pharmaceutical Biotechnology and MOE Key Laboratory of Model Animals for Disease Study, Model Animal Research Center, Nanjing University, 12 Xuefu Rd, Pukou, Nanjing, Jiangsu 210061, China

[2]Institute for Brain Sciences, Nanjing University

Correspondence and requests for materials should be addressed to D.C. (email: chendi@nju.edu.cn; cedauer@gmail.com)

Supplemental Information

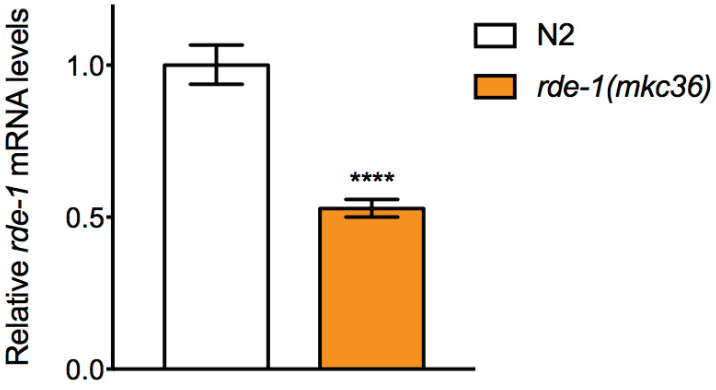

Figure S1. RT-qPCR measurement of *rde-1* mRNA levels in the wild-type N2 and *rde-1(mkc36)* mutant backgrounds. ****, $p < 0.0001$ (n = 3, *t* - tests).

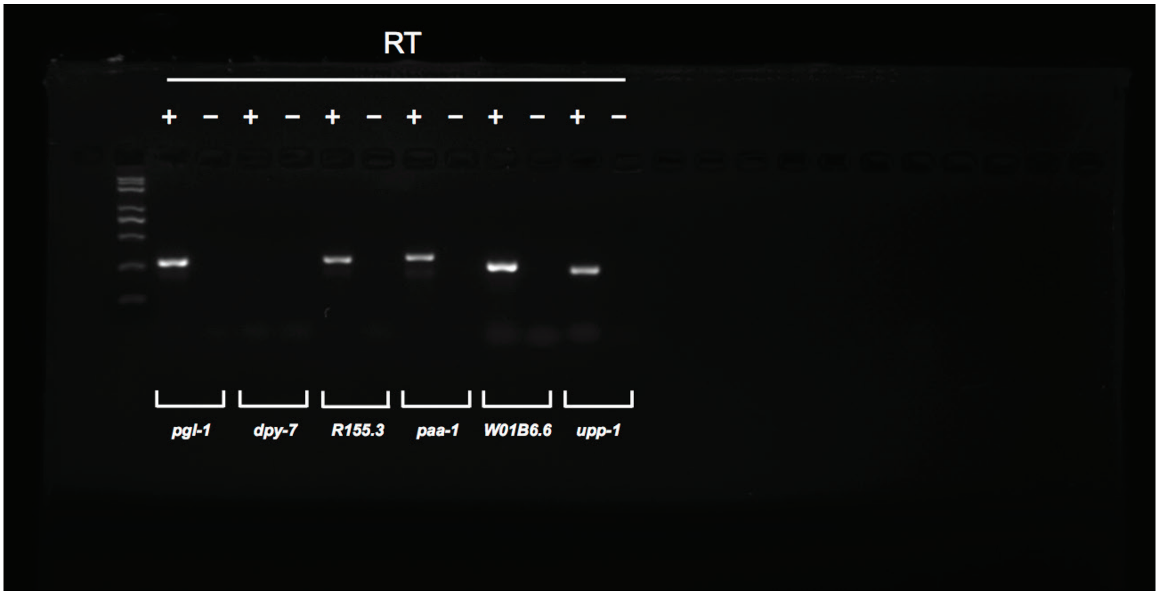

Figure S2. The original gel image of Fig. 5B. RT-PCR products of *R155.3*, *paa-1*, *W01B6.6* and *upp-1* amplified from reverse transcription products using RNAs extracted from dissected gonadal tissues. RT, reverse transcriptase.

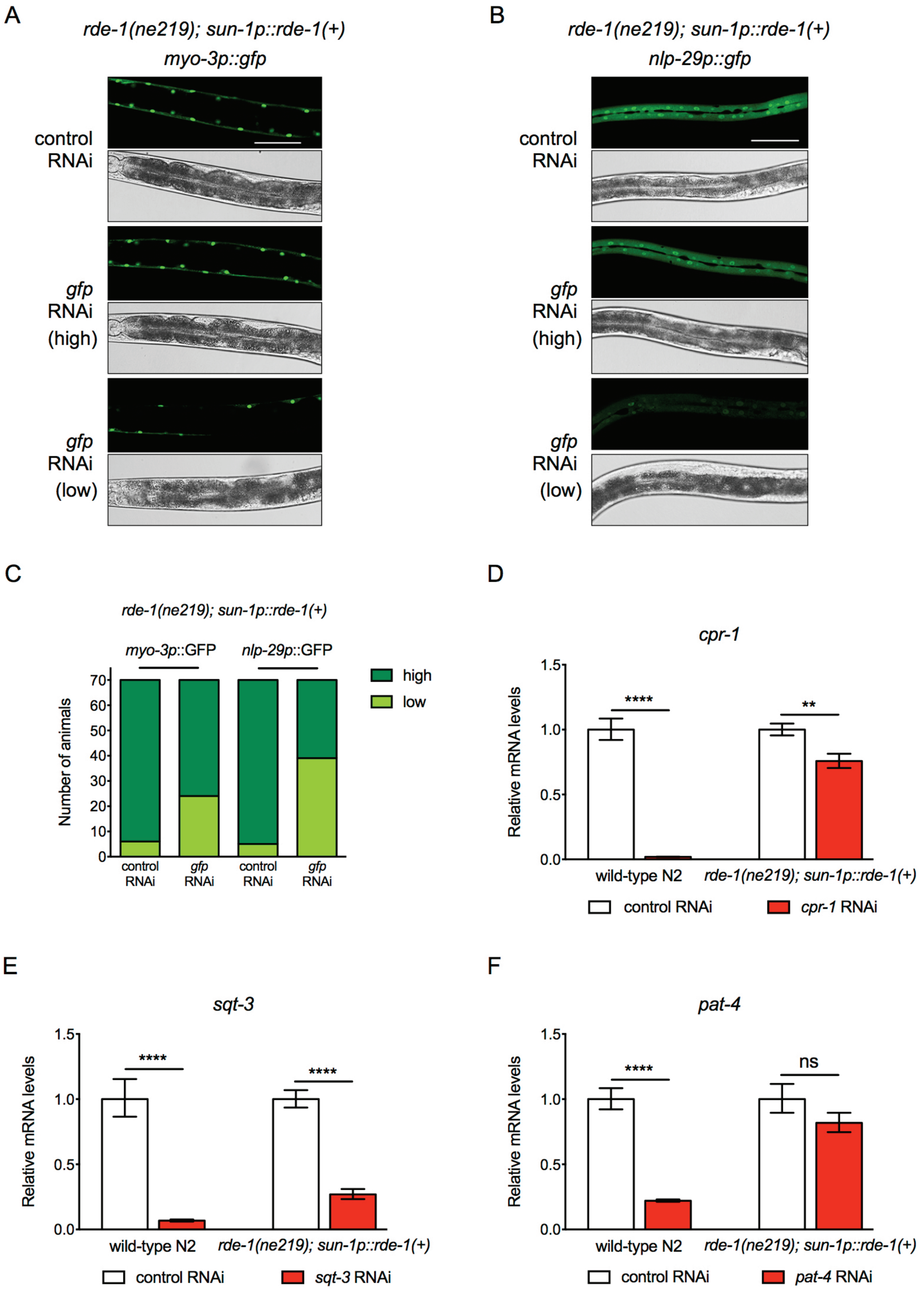
A
rde-1(ne219); sun-1p::rde-1(+)
myo-3p::gfp
control RNAi
gfp RNAi (high)
gfp RNAi (low)
B
rde-1(ne219); sun-1p::rde-1(+)
nlp-29p::gfp
control RNAi
gfp RNAi (high)
gfp RNAi (low)
C
rde-1(ne219); sun-1p::rde-1(+)
myo-3p::GFP
nlp-29p::GFP
Number of animals
0
10
20
30
40
50
60
70
high
low
control RNAi
gfp RNAi
control RNAi
gfp RNAi
D
cpr-1
Relative mRNA levels
0.0
0.5
1.0
1.5
****
**
wild-type N2
rde-1(ne219); sun-1p::rde-1(+)
control RNAi
cpr-1 RNAi
E
sqt-3
Relative mRNA levels
0.0
0.5
1.0
1.5
****
****
wild-type N2
rde-1(ne219); sun-1p::rde-1(+)
control RNAi
sqt-3 RNAi
F
pat-4
Relative mRNA levels
0.0
0.5
1.0
1.5
****
ns
wild-type N2
rde-1(ne219); sun-1p::rde-1(+)
control RNAi
pat-4 RNAi

Figure S3. The DCL484 germline *rde-1* rescue strain shows RNAi activities in the soma. (A-B) Representative fluorescence and bright field images of the muscular *myo-3p::gfp* (A) and epidermal *nlp-29p::gfp* (B) reporters treated with control or *gfp* RNAi in the DCL484 germline *rde-1* rescue strain, which carries the *rde-1(ne219)* mutation. *gfp* RNAi treated animals with high GFP expression (middle panels) showed no leakiness of RNAi effects, whereas *gfp* RNAi treated animals with low GFP expression (lower panels) showed RNAi efficiency in the soma. Scale bar, 50 μm. (C) Quantification of GFP expression. 70 animals were scored in each treatment. (D-F) RT-qPCR measurement of the intestine-specific *cpr-1* (D), epidermis-specific *sqt-3* (E) and muscle-specific *pat-4* (F) mRNA levels in the wild-type and DCL484 germline *rde-1* rescue strain treated with the control or corresponding RNAi. ****, $p < 0.0001$; **, $p < 0.01$; ns, $p > 0.05$ (n = 3, *t* - tests).

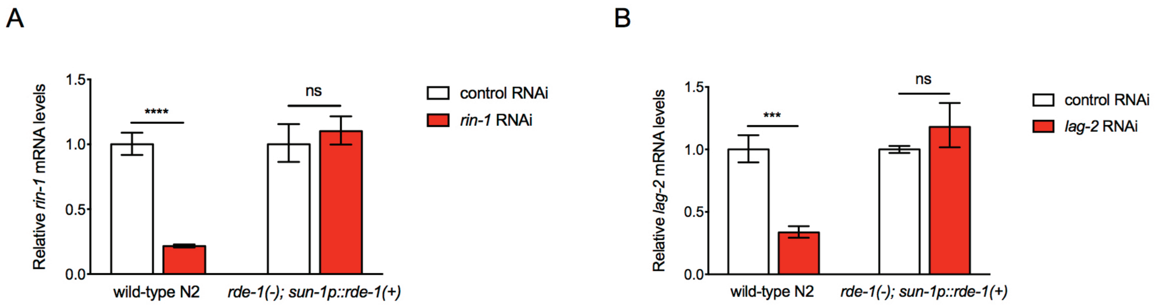

Figure S4. The DCL569 germline *rde-1* rescue strain shows no obvious RNAi efficiency in the somatic gonad. (A-B) RT-qPCR measurement of somatic gonadal *rin-1* (A) and *lag-2* (B) mRNA levels in the wild-type or *rde-1* germline rescue animals treated with the control or corresponding RNAi. ****, $p < 0.0001$; ***, $p < 0.001$; ns, $p > 0.05$ (n = 3, *t* - tests).

Table S1. Sequences of primers

Primer	Sequence
Primers for the *sun-1p::rde-1::sun-1 3'UTR* construct	
*sun-1*p F	GCTACGTAATACGACTATTTTTATTACTAAAATCAGTTTCTAAAATG
*sun-1*p R	TCGGGAAAATTCGAGGACATACCGAGTAGATCTGGAAGTTTAGA
rde-1 F	CCAGATCTACTCGGTATGTCCTCGAATTTTCCCGAATTGG
rde-1 R	ATAATACGGCGTTTTTATGCGAACGACATTCCAGGGTACTTC
sun-1 3′UTR F	GAATGTCGTTCGCATAAAAACGCCGTATTATTGTTCCTGC
sun-1 3′UTR R	ACTTATAATACGACTCACTAGTGAGAAAACAACAGTGGTTTTCTCCTAA
pCFJ151 F	ATGAGAGCCGTCCTTATTGAATGCAAAATCCTTTCAAGCATTCCC
pCFJ151 R	GAAACTGATTTTAGTAATAAAAATAGTCGTATTACACTGTTGTTTTCTCA CTAGTGAGTCGTATTATAAGTGCAAGCGTAGCCCGGGCCTAG
Primers for RT-qPCR	
pgl-1 RTF	CGTCGATAGCTTCAAGAAATTTGTTCAAGG
pgl-1 RTR	TGTATCGGACAAACAACACTTCTCC
daz-1 RTF	CTCCACGCCAAGGCAAGAAGAG
daz-1 RTR	CTCTCAGTGCGAGCTCCTTTGATTGG
cpr-1 RTF	CAAGCACTTCGGAGTCTCTGC
cpr-1 RTR	AGAATGCAGCCTCAACTGGT
sqt-3 RTF	GGGAATCTGTCCAAAGTATTGCGCTC
sqt-3 RTR	ACGCAGAAGTTGAACATCGTCTACC
pat-4 RTF	CCGAACTTGGTGATCATCTCGCAG
pat-4 RTR	CGTGATCAATGACGACAGAACTTTGCTC
rde-1 RTF	AGAAGCGAATCGGAGTTACAAATTCCT
rde-1 RTR	GCGAATTGTACTTTAATCTCCTCGTTCAC
rin-1 RTF	GTACCGTATTCAACGAGCCA
rin-1 RTR	TGGTTCACTGACTTTCAATCCC
lag-2 RTF	CTGCTTGAATGGCGCCAAATGCT
lag-2 RTR	GGCTTGAACTTGTACTTGAAGCATCCGA
Primers for RT-PCR from dissected gonad	
pgl-1 RTF	TGAAACAGTTGATGTTGGATGGTC
pgl-1 RTR	CTGACACTCGTAGGAATCCCA
dpy-7 RTF	GGGCTCCTTCAAGTTCGTTGCTC
dpy-7 RTR	GTCGCCCATTAGCTCCATCCATTCC
R155.3 RTF	TGTTCAACCTGACACTCTATTGTTCACC
R155.3 RTR	GAGCATCGACAAACAATCCAAGCA
paa-1 RTF	TTACCGACCATGTGTTCTCAATCC
paa-1 RTR	GTTCTTTCCAATTCGCTTCAAACTC
W01B6.6 RTF	GATGTTCCCGAGTTCAACCGA
W01B6.6 RTR	ATACGCTGTCATTGCCACTG
upp-1 RTF	TGTGCGCCGACGATTTCTATGAG
upp-1 RTR	GATTTGAACCTGATCTCCATCCATACGA
